# Wheat transcriptome profiling reveals abscisic and gibberellic acid treatments regulate early-stage phytohormone defense signaling, cell wall fortification, and metabolic switches following *Fusarium graminearum*-challenge

**DOI:** 10.1186/s12864-021-08069-0

**Published:** 2021-11-06

**Authors:** Leann M. Buhrow, Ziying Liu, Dustin Cram, Tanya Sharma, Nora A. Foroud, Youlian Pan, Michele C. Loewen

**Affiliations:** 1 National Research Council of Canada, Aquatic and Crop Resources Development Research Centre, 110 Gymnasium Place, Saskatoon, SK S7N 0M8 Canada; 2 National Research Council of Canada, Digital Technologies Research Centre, 1200 Montreal Road, Ottawa, ON K1A 0R6 Canada; 3 University of Ottawa, Department of Chemistry and Biomolecular Sciences, 150 Louis-Pasteur Pvt, Ottawa, ON K1N 6N5 Canada; 4 Agriculture and Agri-food Canada, Lethbridge Research and Development Centre, 5403 1st Ave, Lethbridge, AB T1J 4B1 Canada; 5 National Research Council of Canada, Aquatic and Crop Resources Development Research Centre, 100 Sussex Drive, Ottawa, ON K1A 0R6 Canada

**Keywords:** Wheat, *Triticum aestivum*, *Fusarium graminearum*, Fusarium head blight, Phytohormone, Abscisic acid, Gibberellic acid, Differentially expressed genes, RNA-seq

## Abstract

**Background:**

Treatment of wheat with the phytohormones abscisic acid (ABA) and gibberellic acid (GA) has been shown to affect Fusarium head blight (FHB) disease severity. However, the molecular mechanisms underlying the elicited phenotypes remain unclear. Toward addressing this gap in our knowledge, global transcriptomic profiling was applied to the FHB-susceptible wheat cultivar ‘Fielder’ to map the regulatory responses effected upon treatment with ABA, an ABA receptor antagonist (AS6), or GA in the presence or absence of *Fusarium graminearum* (*Fg*) challenge.

**Results:**

Spike treatments resulted in a total of 30,876 differentially expressed genes (DEGs) identified in ‘Fielder’ (26,004) and the *Fg* (4872) pathogen. Topology overlap and correlation analyses defined 9689 wheat DEGs as *Fg*-related across the treatments. Further enrichment analyses demonstrated that these included expression changes within ‘Fielder’ defense responses, cell structural metabolism, molecular transport, and membrane/lipid metabolism. Dysregulation of ABA and GA crosstalk arising from repression of ‘Fielder’ *FUS3* was noted. As well, expression of a putative *Fg* ABA-biosynthetic cytochrome P450 was detected. The co-applied condition of *Fg* + ABA elicited further up-regulation of phytohormone biosynthesis, as well as SA and ET signaling pathways and cell wall/polyphenolic metabolism. In contrast, co-applied *Fg* + GA mainly suppressed phytohormone biosynthesis and signaling, while modulating primary and secondary metabolism and flowering. Unexpectedly, co-applied *Fg* + AS6 did not affect ABA biosynthesis or signaling, but rather elicited antagonistic responses tied to stress, phytohormone transport, and FHB disease-related genes.

**Conclusions:**

Observed exacerbation (misregulation) of classical defense mechanisms and cell wall fortifications upon ABA treatment are consistent with its ability to promote FHB severity and its proposed role as a fungal effector. In contrast, GA was found to modulate primary and secondary metabolism, suggesting a general metabolic shift underlying its reduction in FHB severity. While AS6 did not antagonize traditional ABA pathways, its impact on host defense and *Fg* responses imply potential for future investigation. Overall, by comparing these findings to those previously reported for four additional plant genotypes, an additive model of the wheat-*Fg* interaction is proposed in the context of phytohormone responses.

**Supplementary Information:**

The online version contains supplementary material available at 10.1186/s12864-021-08069-0.

## Background

Fusarium head blight (FHB), one of the more prevalent diseases of wheat (*Triticum aestivum* L.), is the result of infection of wheat heads by the hemi-biotrophic ascomycetous *Fusarium graminearum* (*Fg*) and related species [1]. FHB remains a significant pathogenic threat to the agricultural industry; *Fg* infection decreases grain yields and deposits mycotoxins [2, 3]. Wheat breeding programs aimed at addressing FHB have resulted in incremental benefits to date [4], while chemical control measures remain limited by host-developmental requirements of the pathogen [5]. Thus, it is imperative that we expand our understanding of these host-pathogen interactions to identify new sources of resistance and antifungal molecular targets.

The transcriptomic responses of FHB-susceptible and -resistant wheat varieties in response to *Fg* challenge have been investigated extensively over the course of the last decade (reviewed in [6]; and more recently [7–11]). Together these studies highlight a breadth of host-responses that include modulation of primary metabolism and photosynthesis, transcriptional and translational regulators, traditional plant defense responses (both phytohormone-associated and pathogenesis-related protein targets), and detoxification genes. The role of the classical defense phytohormones, salicylic acid (SA), jasmonic acid (JA), and ethylene (ET) in the wheat defense response to *Fg*-challenge has been extensively described with a consensus model of early biotrophic SA followed by later stage necrotrophic JA/ET responses [6, 8, 9]. Nonetheless, the role of ET remains in question, with conflicting reports highlighting mediation of both resistance [3] and susceptibility [12] in early and late responses [9].

In agreement with independent research groups investigating other wheat varieties and FHB disease stages [9, 13, 14], our previous report described drastic alterations of phytohormone profiles in the FHB-susceptible *T. aestivum* cultivar ‘Fielder’ when challenged with *Fg* [15]. Two such FHB-regulated phytohormones, when co-applied with pathogen challenge, modulated disease severity and spread where abscisic acid (ABA) promoted infection and gibberellic acid (GA) reduced infection [14, 15]. It has been established that *Fusarium* spp. can themselves produce ABA [14], GA [16], auxin (indole acetic acid; IAA [17]), and cytokinins (CK [18];), while also encoding both 1-aminocyclopropane carboxylic acid (ACC) synthases and deaminases potentially involved in ET biosynthesis [19]. Therefore, although phytohormones may be traditionally thought to serve as plant host signaling and defense molecules, *Fusarium* spp*.* may hijack or dysregulate phytohormone metabolism to establish or promote infection.

Whether exogenously applied, or derived from wheat or *Fusarium*, the mechanisms by which ABA and GA alter host resistance and susceptibility during *Fg* infection remains unclear. As ABA and GA antagonistically regulate many plant developmental processes and elicit opposing changes to ‘Fielder’ FHB disease, it is tempting to hypothesize that these phytohormones may dysregulate each other’s biosynthesis, signaling, and crosstalk with classical defense phytohormones. In the present study, this hypothesis is largely discounted as robust phytohormone-related gene expression antagonism is not observed. Instead, more than 30,000 DEGs are identified across both wheat and *Fg* genomes in response to the seven combinatorial treatment types. To deconvolute this complex data set, differential expression feature extraction (DEFE) was employed, ultimately allowing DEGs highly enriched upon *Fg* challenge to be identified and further analyzed for association networks across treatments. Subsequently, the DEGs elicited by *Fg* challenge, ABA or GA treatment alone, and the combined effects of both the pathogen and phytohormone were individually targeted. This analysis also considers DEGs arising upon co-application of the antagonist of ABA receptors, AS6 [20]. Ultimately, transcriptomic responses of five wheat genotypes upon *Fusarium* challenge are compared to further contribute to a consensus model of this plant-pathogen interactions. We discuss how transcriptomic changes elicited by ABA and GA impact such a consensus model and may contribute to the modulation of FHB symptoms previously reported in Qi et al. [14] and Buhrow et al. [15].

## Results

### Transcriptome overview

**Fig. 1:**
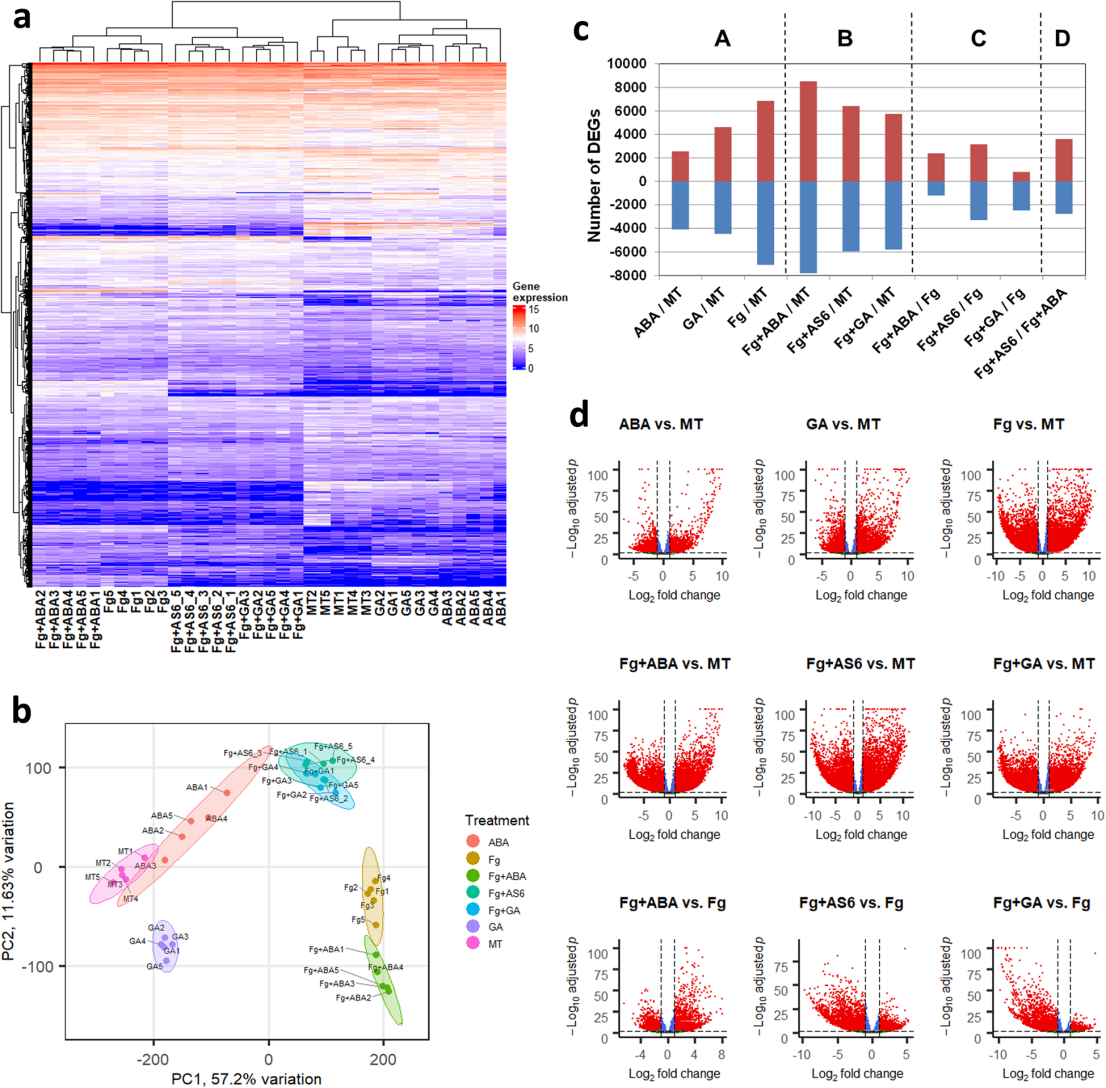
Transcriptome overview. Gene expression levels were log_2_ transformed read counts. **a** heatmap of wheat DEGs; **b** PCA of wheat DEGs; **c** wheat DEG distribution over the 10 pairwise comparisons, A: “alone” conditions compared with MT, B: co-applications compared with MT, C: co-applications compared with *Fg* alone, D: comparison between two co-applications; **d** volcano plots of wheat DEGs of the first nine pairwise comparisons (sub-panel A, B, C of panel d), adjusted *p* values less than 10^− 100^ were displayed as 10^− 100^

FHB-susceptible wheat cultivar ‘Fielder’ spikes were treated with ABA (condition: ABA), GA (condition: GA), or *Fg* (condition: *Fg*) alone. Additionally, ‘Fielder’ spikes were treated with a combination of *Fg* and ABA (condition: *Fg* + ABA), GA (condition: *Fg* + GA) or an ABA receptor antagonist (condition: *Fg* + AS6). RNA-seq reads (Additional file 1 Tab ‘S1’) were remapped to a combination of the wheat genome (IWGSC RefSeq v1.0 [21];) and *Fusarium graminearum* (str. PH-1) resulting in 97% of the reads being successfully mapped to the two reference genomes (Additional file 1, Tab ‘S1’). *Fg*-challenge and phytohormone application resulted in consistent and distinguishable changes to the ‘Fielder’ transcriptome (Fig. 1). After normalization of the read counts, a total of 30,876 differentially expressed genes (DEGs) were identified based on the criteria specified in the Methods section, including 4872 from *F. graminearum* and 26,004 from wheat (Table 1; Additional files 2 & 3).

**Table 1:** Number of DEGs arising from each pairwise comparison. For each treatment condition and appropriate reference background, mock-treated (MT) or *Fg* challenged (*Fg*), the total number of DEGs are presented. DEGs are further subdivided based on mapping to the wheat or *Fg* genome and directionality of the expression changes (up-regulated or down-regulated)

		Condition									
DEG Population	Regulation	ABA/MT	GA/MT	***Fg***/MT	***Fg + ABA***/MT	***Fg + AS6***/MT	***Fg + GA***/MT	***Fg + ABA***/***Fg***	***Fg + GA***/***Fg***	***Fg + AS6***/***Fg***	***Fg + AS6***/***Fg + ABA***
**Total**	**Up**	2566	4623	8691	11772	7668	10560	2477	1703	3163	3602
	**Down**	4098	4473	7100	7810	5962	5798	1213	2472	3325	3330
**Wheat**	**Up**	2566	4623	6855	8529	6410	5743	2398	806	3146	3602
	**Down**	4098	4473	7100	7810	5962	5798	1213	2471	3299	2773
***Fg***	**Up**	0	0	1836	3243	1258	4817	79	897	17	0
	**Down**	0	0	0	0	0	0	0	1	26	557

Toward assessing the relative effects of the different treatments, differential expression feature extraction (DEFE [22];) analysis was performed. Among the 729 (3^6^) theoretically possible ‘M’ DEFE-patterns for the six treatments as they compared with mock water treatment (MT), 266 had one or more genes. A list of 30 highly populated DEFE patterns collectively contain 17,170 (66%) of the wheat DEGs and 4839 (99%) of the *Fg* DEGs (Table 2). The frequency distribution in other DEFE patterns is available in Additional files 2 & 3 Tab ‘DEFE_stats’. Observations of the patterns containing the highest number of wheat DEGs included those up- or down-regulated by the pathogen irrespective of one or more coapplied compounds [M001100 (1736 DEGs), M202222 (1641 DEGs), M001111 (1593 DEGs), M002222 (1181 DEGs)]. At a high level, the DEFE and principal component analyses (PCA discussed in next section; Fig. 1b) supported the fact that a high number of DEGs were derived from pathogen challenge, while fewer DEGs were derived from ABA or GA treatment. Similarly, *Fg* DEGs were highly enriched with four DEFE patterns, which collectively accounted for 97% of *Fg* DEGs (Table 1, Additional file 3).

**Table 2:** Number of genes in highly informative Differential Expression Feature Extraction (DEFE) patterns M (ABA/MT, GA/MT, *Fg*/MT, *Fg* + ABA/MT, *Fg* + AS6/MT, *Fg* + GA/MT)

Pattern	Wheat genes	FHB related wheat genes^a^	*Fg* genes	Description
M000001	289	41%	1589	up-regulated by *Fg + GA*, but not by them separately
M000002	111	31%	0	down-regulated by *Fg + GA*, but not by them separately
M000010	994	28%	6	up-regulated by *Fg + AS6,* but not by them separately
M000020	248	37%	0	down-regulated by *Fg + AS6,* but not by them separately
M000100	698	19%	40	up-regulated by *Fg + ABA*, but not by them separately
M000200	960	21%	0	down-regulated by *Fg + ABA*, but not by them separately
M001000	378	44%	5	up-regulated by *Fg* alone
M002000	429	39%	0	down-regulated by *Fg* alone
M010000	1306	4%	0	up-regulated by GA alone
M020000	1163	3%	0	down-regulated by GA alone
M100000	254	7%	0	up-regulated by ABA alone
M200000	218	3%	0	down-regulated by ABA alone
M001111	1593	**97%**	1165	up-regulated by *Fg*, with or without a hormone
M002222	1181	**94%**	0	down-regulated by *Fg*, with or without a hormone
M000111	309	**77%**	72	up-regulated by *Fg* in combination of any of the three hormones
M000222	29	**55%**	0	down-regulated by *Fg* in combination of any of the three hormones
M010001	10	0%	0	up-regulated by GA, with or without *Fg*
M020002	10	0%	0	down-regulated by GA, with or without *Fg*
M100100	9	0%	0	up-regulated by ABA, with or without *Fg*
M200200	22	0%	0	down-regulated by ABA, with or without *Fg*
M111111	750	42%	0	up-regulated by all treatments
M222222	964	42%	0	down-regulated by all treatments
M001100	1736	32%	0	up-regulated by *Fg*, but GA or AS6 neutralized/suppressed the effect of *Fg*
M002200	543	38%	0	down-regulated by *Fg*, but GA and AS6 neutralized/suppressed the effect of *Fg*
M000101	45	58%	1315	up-regulated by *Fg* in combination of either ABA or GA
M000202	27	63%	0	down-regulated by *Fg* in combination of either ABA or GA
M001101	552	81%	647	up-regulated by *Fg* with or without combination of either ABA or GA
M002202	323	73%	0	down-regulated by *Fg* with or without combination of either ABA or GA
M101111	378	70%	0	up-regulated by all treatment excepting GA alone
M202222	1641	86%	0	down-regulated by all treatment excepting GA alone

^a^Percentage of wheat genes with this DEFE pattern were classified as FHB related genes. This indicates the worth noting importance of a differential expression profile

### Wheat DEGs highly correlated with *Fg* challenge

Wheat transcriptomic responses were most clearly divided based on the presence or absence of *Fg* challenge, contributing 57.2% of the variance in the PCA (Fig. 1b). Through topology overlap analysis using WGCNA [23], 58 DEG clusters were identified in this dataset with 17 being significantly correlated (*p* < 5 × 10^− 2^; 12,138 DEGs) to *Fg* treatment (Additional file 2 Tab ‘WheatDEGs’ @Col-M,N & Tab ‘moduleTraitCor’). This finding was further validated by correlation analysis which revealed 10,609 individual *Fg*-correlated DEGs. Of these, 9689 (91%) were common to the *Fg* correlated topology overlap clusters and were thus considered ‘*Fg*-related genes’ (Additional file 2 Tab ‘WheatDEGs’ @Col-O). The majority (83%) of these 9689 *Fg*-related ‘Fielder’ genes fall into the most informative ‘M’ DEFE patterns listed in Table 2.

To further understand the relationship of DEGs identified by topology overlap, gene association network analysis was performed using the WGCNA R package [23]. The top 1% of the topology overlap matrix was considered in the entire network consisting of 10,373 wheat DEGs, connected by 3,370,764 edges. Of these, 8349 (80%) were *Fg*-related genes. The top 2621 genes having connection degrees of 1000 or higher were considered to be potential key regulators of the associated group of genes, and are referred to as key hub genes (highlighted in Additional file 2 Tab ‘WheatDEGs’@Col-P). An enrichment index analysis of gene groups was performed on the network genes as described in Pan et al., [8]. Among the highly enriched group of genes in the network were alkaline shock protein 23, clathrin assembly family protein, D-glycerate dehydrogenase / hydroxypyruvate reductase, photosystem II 22 kDa, chloroplastic, PISTILLATA-like MADS-box transcription factor, fatty acid hydroxylase, fimbrin-like protein 2, pollen allergen, glyoxal oxidase, jasmonate ZIM domain proteins, C2 domain-containing protein, yellow stripe-like transporters, and pectinesterase (Additional file 4). The B3 domain-containing transcription factor *FUS3* gene (TraesCS3D01G249100) was among the key hub genes, with 2281 immediate neighborhood genes in the network. It was significantly down-regulated by all conditions that include *Fg*-treatment (adj *p* < 2 × 10^− 14^) (Additional file 5: Figure S1). Interestingly, all conditions that included *Fg*-treatment also expressed a putative fungal ABA biosynthetic cytochrome P450 homolog (FGRAMPH1-01 T26277, Additional file 5: Figure S2B).

### ABA and GA treatments alone elicited diverse metabolic changes to the wheat transcriptome

**Fig. 2:**
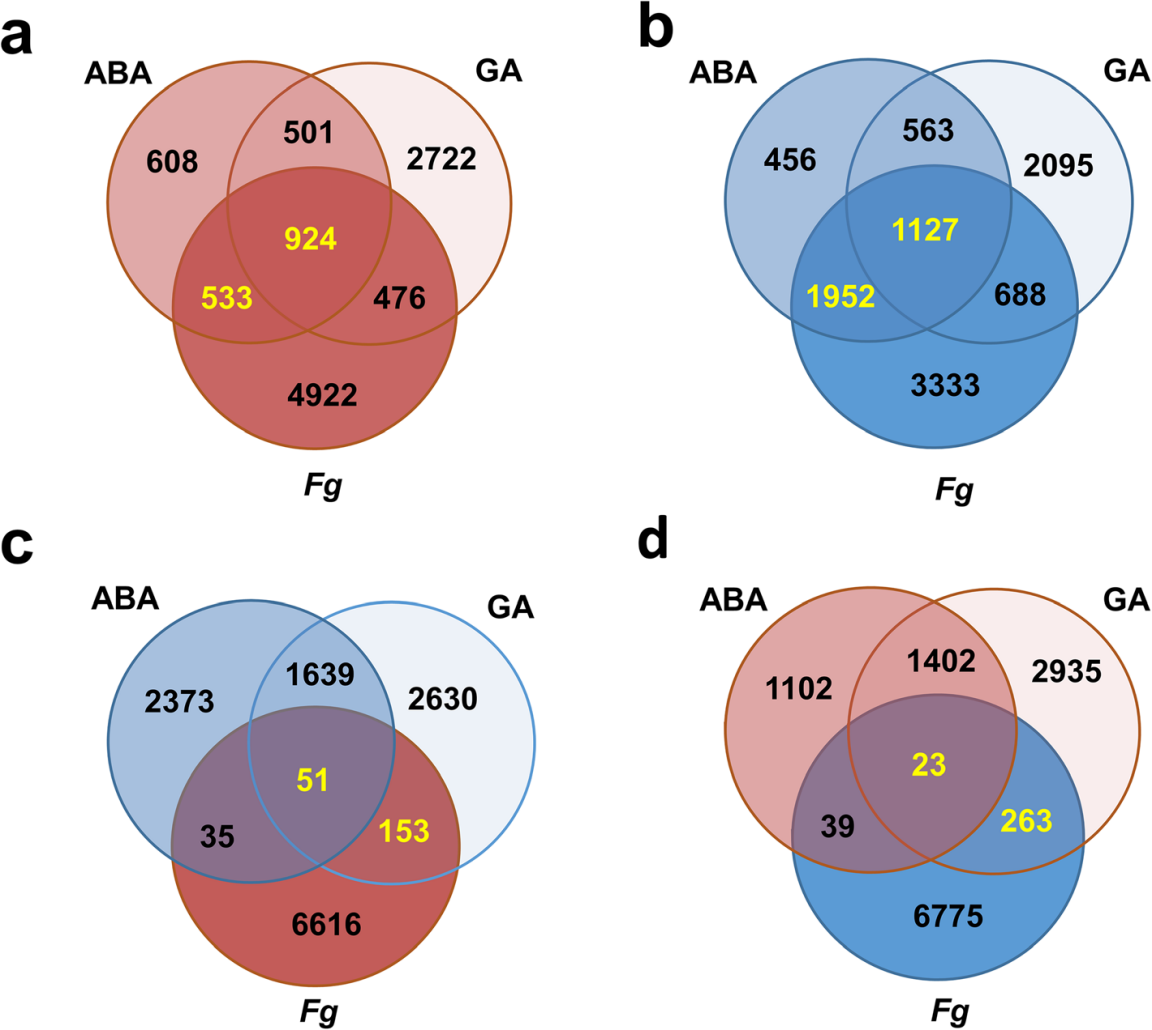
Share of *Fg* alone up- or down- regulated genes that are up- and down- regulated under ABA and GA alone conditions, with all DEGs calculated compared to mock treated (MT). In all cases, up-regulated genes are highlighted in shades of red, down-regulated genes are highlighted in shades of blue. DEGs called in text are yellow. **a** Up-regulated DEGS. **b** Down-regulated DEGS. Opposing DEGs: **c** up-regulated by *Fg* but down-regulated by ABA and GA; **d** down-regulated by *Fg* but up-regulated by ABA and GA

To characterize the impacts of ABA and GA application alone, these data were compared to MT. Neither phytohormone treatment elicited wheat responses as strong as *Fg* challenge (Fig. 1a & b; Table 1); however, ABA down-regulated nearly twice as many wheat genes as it up-regulated, while GA modified approximately equal numbers of genes up and down (Table 1, Fig. 1c & d), consistent with the hormone-responsive trends recently reported on the FHB-susceptible wheat cultivar ‘Roblin’ [11]. ABA treatment most notably led to down-regulation of 3079 (43%) wheat DEGs that were down-regulated by *Fg* as well as up-regulation of 1457 (21%) wheat DEGs that were up-regulated by *Fg* (Fig. 2). Furthermore, ABA elicited few opposing effects when compared to the *Fg* condition (GO enrichment of these DEGs found in Additional file 6 Tab ‘FU2’). GA treatment elicited 20–25% of wheat DEGs in the same directionality as the *Fg* condition, particularly down-regulated 204 (3%) DEGs that were up-regulated by *Fg* and conversely up-regulating 286 (4%) DEGs that were down-regulated by *Fg* (Fig. 2; GO enrichment of these DEGs found in Additional file 6 Tab ‘FU4’).

### *Fg* challenge with ABA and GA co-application respectively enhanced and suppressed wheat DEGs elicited by *Fg* alone

The wheat transcriptomic changes elicited by ABA (*Fg* + ABA) and GA (*Fg* + GA) co-application in the presence of *Fg* pathogen challenge were collectively analyzed as compared to MT. This enabled a direct comparison of gene regulation events mediated by phytohormones alone to those arising from the condition where all three components of the ‘plant-pathogen-phytohormone’ interaction were present together. In the case of responses common to the *Fg*, *Fg* + ABA and *Fg* + GA treatments (*Fg*∩[*Fg* + ABA]∩[*Fg* + GA]), *Fg* + ABA and *Fg* + GA jointly up-regulated 3751 (55%) wheat DEGs that were up-regulated by *Fg* alone and down-regulated 4696 (66%) DEGs that were down-regulated by *Fg* alone (highlighted in Additional file 5: Figure S3A & B). The co-applications also elicited opposing wheat DEGs; *Fg* + ABA up-regulated 63 and down-regulate 4 DEGs, and *Fg* + GA up-regulated 2 and down-regulated 5 DEGs that were down-regulated and up-regulated by *Fg* alone (highlighted in Additional file 5: Figure S3C & D). These changes, along with non-overlapping DEGs, manifested in an increase upon *Fg* + ABA and slight reduction upon *Fg* + GA treatments in the overall DEG counts when compared to *Fg* alone (Fig. 1c; Table 1). When further considering DEGs elicited by the phytohormone, pathogen, and co-applied conditions, the overlap in elicited wheat transcriptomic changes was over 90% DEGs common in the case of ABA (ABA∩*Fg*∩[*Fg* + ABA]) and over 80% DEGs common in the case of GA (GA∩*Fg*∩[*Fg* + GA]; Additional file 5: Figure S4).

**Fig. 3:**
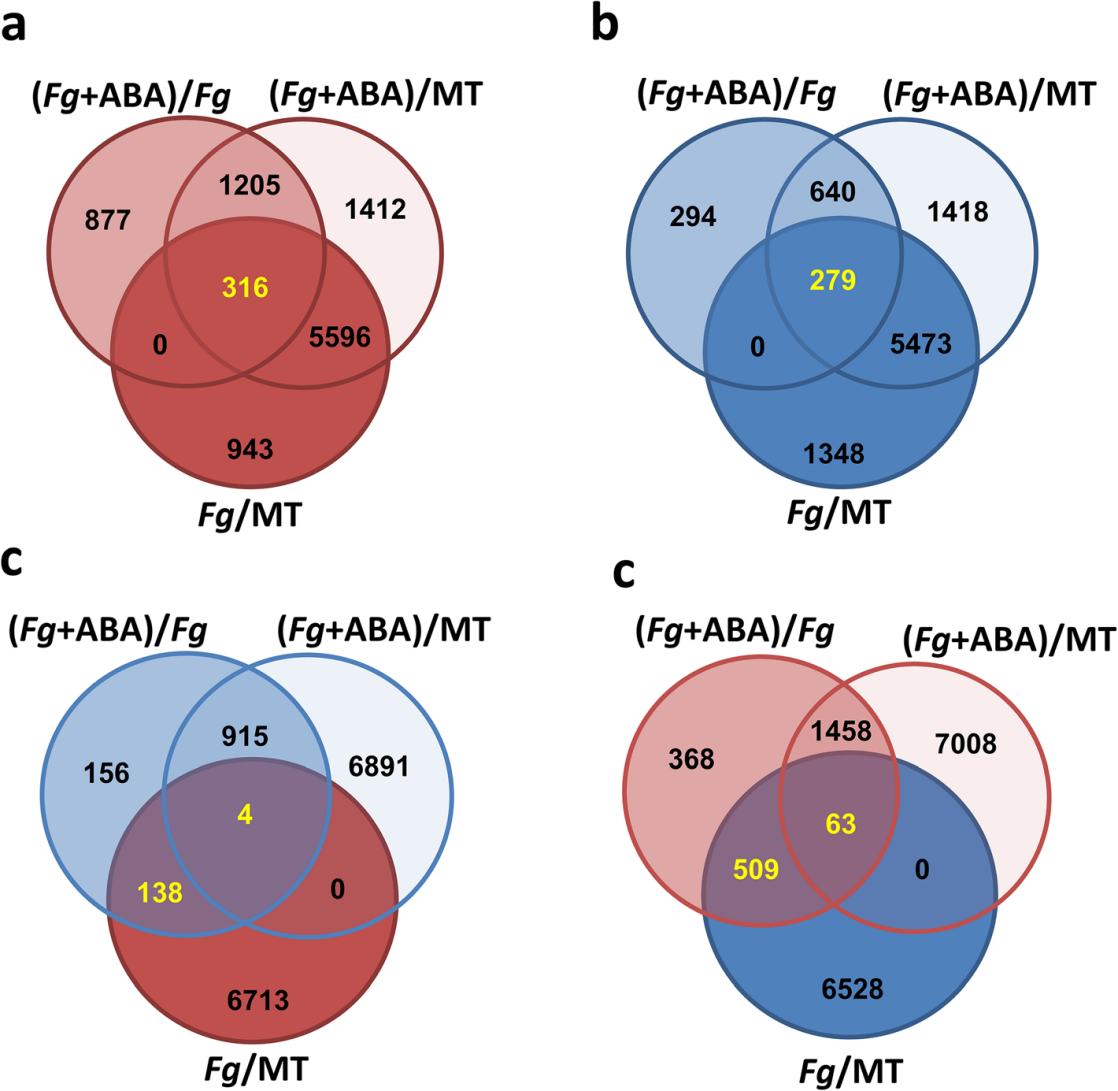
Comparison of gene regulation elicited by ABA in the presence of *Fg*, with DEGs calculated compared to mock treated (MT) as well as to *Fg*. In all cases, up-regulated genes are highlighted in shades of red, and down-regulated genes are highlighted in shades of blue. DEGs called in text are yellow. **a** Distribution of all up-regulated genes by *Fg* + ABA as compared to *Fg*, or *Fg* + ABA and *Fg*-alone compared to MT. **b** Distribution of all down-regulated genes by *Fg* + ABA as compared to *Fg*, or *Fg* + ABA and *Fg*-alone compared to MT. **c** Distribution of up-regulated genes by *Fg*-alone, but down-regulated by *Fg* + ABA as they compared to *Fg* or to MT. **d** Distribution of all down-regulated genes by *Fg*-alone, but up-regulated by *Fg* + ABA as they compared to *Fg* or to MT.

**Fig. 4:**
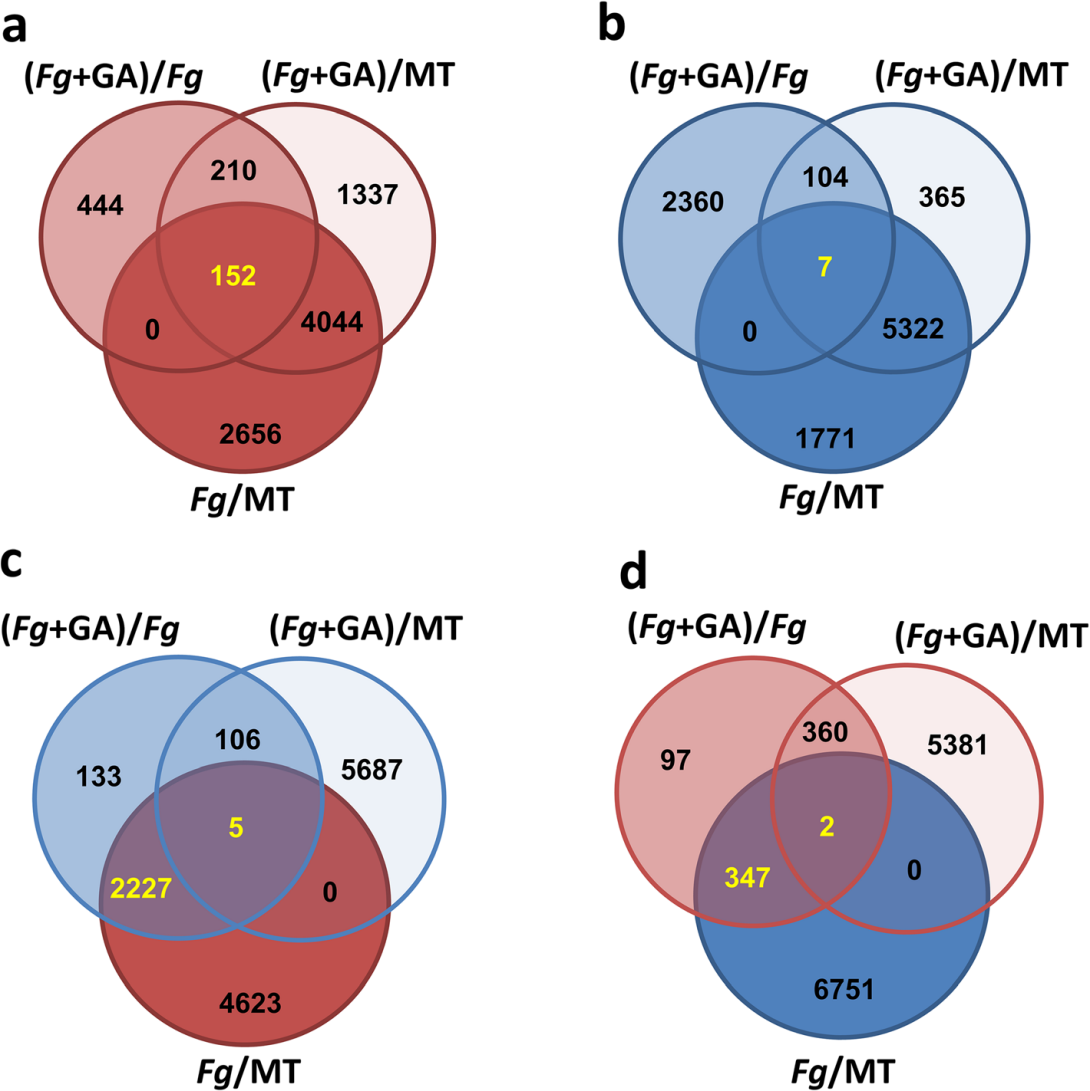
Comparison of gene regulation elicited by GA in the presence of *Fg*, with DEGs calculated compared to mock treated (MT) as well as *Fg*. In all cases, up-regulated genes are highlighted in shades of red, and down-regulated genes are highlighted in shades of blue. DEGs called in text are yellow. **a** Distribution of all up-regulated genes by *Fg* + GA as compared to *Fg*, or *Fg* + GA and *Fg*-alone compared to MT. **b** Distribution of all down-regulated genes by *Fg* + GA as compared to *Fg*, or *Fg* + GA and *Fg*-alone compared to MT. **c** Distribution of up-regulated genes by *Fg*-alone, but down-regulated by *Fg* + GA as they compared to *Fg* or to MT. **d** Distribution of all down-regulated genes by *Fg*-alone, but up-regulated by *Fg* + GA as they compared to *Fg* or to MT.

A second DEFE analysis (‘F’ DEFE-patterns)’ was carried out as compared to *Fg* challenge to describe the additive DEGs elicited upon phytohormone co-application (Table 3; Additional file 2 Tab ‘DEFE_stat’). The *Fg* + ABA co-application significantly enhanced 316 up-regulated and 572 down-regulated *Fg*-elicited wheat DEGs (Fig. 3a & d) while also suppressing 142 up-regulated and 279 down-regulated *Fg*-elicited wheat DEGs (Fig. 3c & b). The *Fg* + GA co-application significantly enhanced 152 up-regulated and 349 down-regulated *Fg*-elicited wheat DEGs (highlighted in Fig. 4a & d) while also suppressing 2232 (33%) up-regulated and 7 down-regulated *Fg*-elicited wheat DEGs (highlighted in Fig. 4c & b). These findings clearly demonstrate the additive effects of *Fg* + ABA and the decremental effects of *Fg* + GA on the ‘Fielder’ transcriptome challenged with *Fg* consistent with the observations that ABA co-application predominantly enhances, and GA predominantly represses, the wheat transcriptome (Fig. 1d).

**Table 3:** Number of genes with highly informative DEFE patterns on enhancement or suppression of a hormone in co-application with *Fg*: F (*Fg* + ABA_vs_*Fg*, *Fg* + AS6_vs_*Fg*, *Fg* + GA _vs_*Fg*)

Pattern	Wheat	***Fg***	Description
F001	212	856	Enhanced by GA
F002	300	1	Suppressed by GA
F010	2046	6	Enhanced by AS6
F020	1036	24	Suppressed by AS6
F100	1639	39	Enhanced by ABA
F200	1038	0	Suppressed by ABA
F011	381	2	Enhanced by both AS6 and GA, but not by ABA
F022	2134	0	Suppressed by both AS6 and GA, but not by ABA
F101	63	31	Enhanced by both ABA and GA, but not by AS6
F202	14	0	Suppressed by both ABA and GA, but not by AS6
F110	548	3	Enhanced by both ABA and AS6, but not GA
F220	93	0	Suppressed by both ABA and AS6, but not by GA
F111	123	6	Enhanced by all three hormones
F222	13	0	Suppressed by all three hormones
F012	1	0	Enhanced by AS6, but suppressed by GA
F021	1	2	Suppressed by AS6, but enhanced by GA
F102	3	0	Enhanced by ABA, but suppressed by GA
F201	8	0	Suppressed by both ABA, but enhanced by GA
F120	16	0	Enhanced by ABA, but suppressed by AS6
F210	29	0	Suppressed by both ABA, but enhanced by AS6
F122	6	0	Enhanced by ABA, but suppressed by both AS6 and GA
F211	18	0	Suppressed by ABA, but enhanced by both AS6 and GA

### Application of ABA and GA elicited wheat DEGs mapped to chromosome 6BL consistent with their modulation of later-stage FHB phenotype

**Fig. 5:**
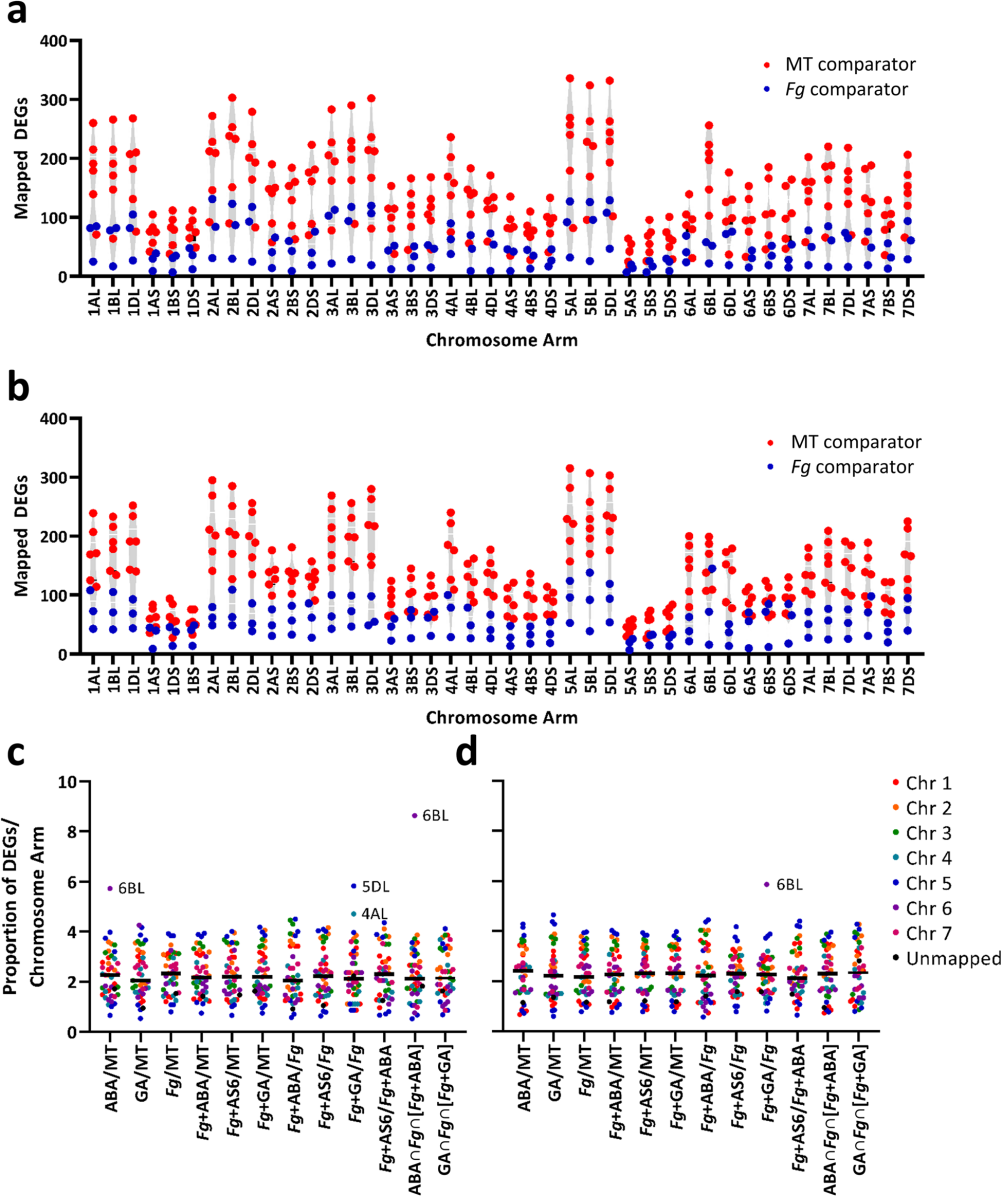
Chromosomal mapping of wheat DEGs elicited by *Fg* and phytohormone treatments. Mapped DEGs as a function of chromosome arm were subdivided into **a** up-regulated and **b** down-regulated DEGs with data points representing individual treatments of *Fg*, phytohormone, or the combination as compared to MT (6 comparisons in red) or *Fg* (3 comparisons in blue). The proportion of DEGs **c** up-regulated or **d** down-regulated was also calculated as DEGs per chromosome arm compared to total and was represented as a function of individual treatment and further parsed by chromosome

**Fig. 6:**
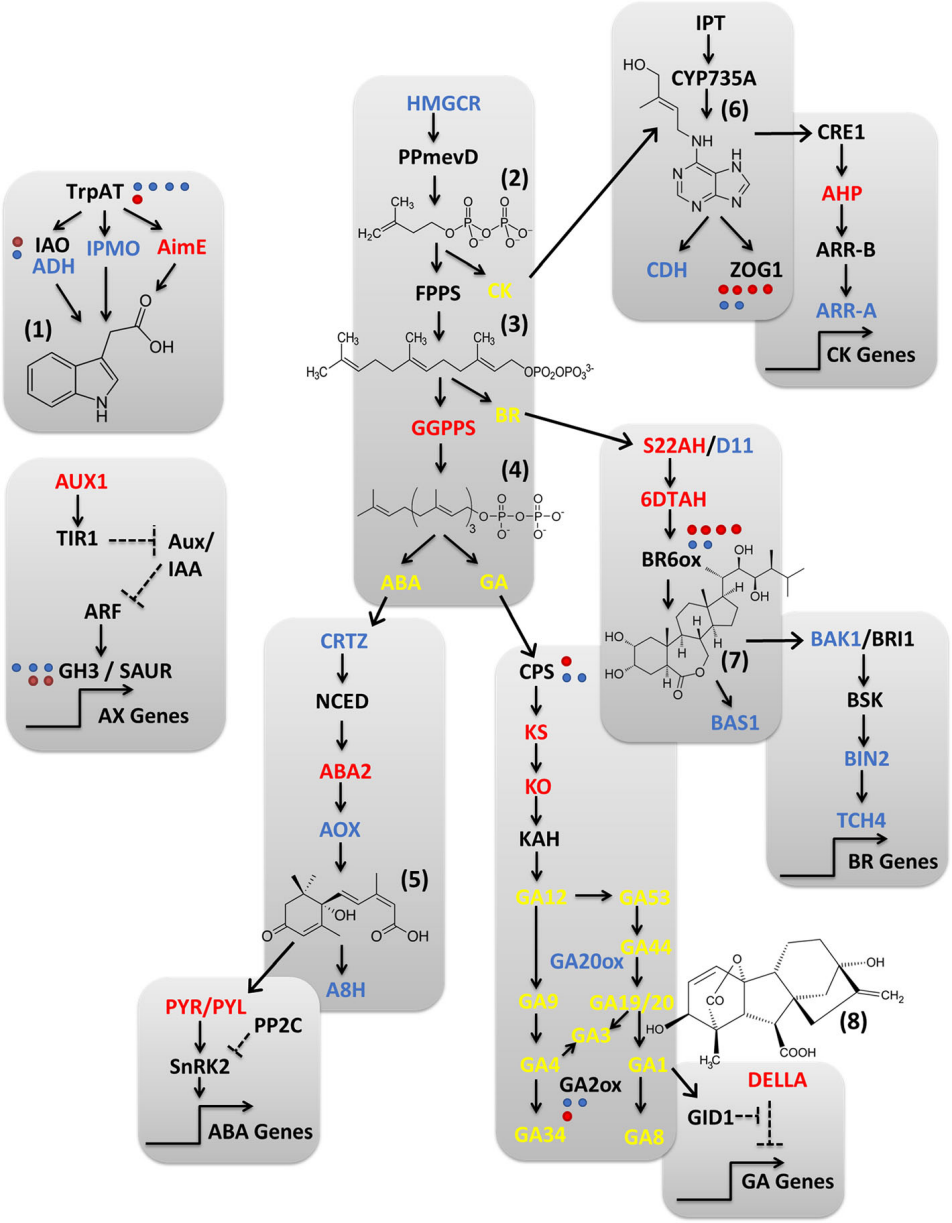
Effect of *Fg* infection on expression of other (non-classical defense) ‘Fielder’ phytohormone biosynthesis and signaling pathway genes. Chemical structures are shown for (1) indole acetic acid, (2) isopentenyl diphosphate (3) farnesyl diphosphate (4) geranylgeranyl diphosphate (5) abscisic acid (6) trans-zeatin (7) brassinolide and (8) giberellin (GA3). Compound acronyms are highlighted in yellow. ‘Fielder’ gene acronyms are represented as up-regulated (red; log_2_FC > 1.5) and down-regulated (blue; log_2_FC < − 1.5), respectively (adj *p* < 0.01) compared to mock treatment. Annotations were based on BlastP analysis selecting for transcripts with > 50% sequence identity to known phytohormone signaling pathway members as annotated in the KEGG database. See Additional file 1, Tabs S2 and S3 for detailed expression data

To understand if the observed modulation of FHB spread by ABA and GA might be tied to a characterized FHB-resistance quantitative trait locus (QTL), DEGs from all treatments were mapped and binned based on chromosomal arm (Fig. 5a & b). Density as a function of physical length [21] was relatively monodisperse with 26.1 ± 6.4 (average ± one standard deviation) up-regulated DEGs and 25.9 ± 6.3 down-regulated DEGs calculated per Mb, excluding Chr5AS for which fewer induced DEGs were mapped (Additional file 5: Figure S5). DEGs appear to map uniformly between the A, B, and D genomes with the exceptions of up-regulated DEGs of Chr 4AL, 5AS, 6BL, and 7BS and down-regulated DEGs of Chr 4AL and Chr 7BS compared to their homoeologs (Fig. 5a & b). Chr 6BL exhibited differential trends with an over-representation of induced DEGs in ABA and ABA∩*Fg*∩[*Fg* + ABA] treatment groups, while also having an over-representation of down-regulated DEGs upon *Fg* + GA treatment. As these early-stage results are in agreement with the late-stage FHB phenotypes elicited by these treatments, it is possible that the genetic underpinnings may be partially attributed to genes located on Chr 6BL. In wheat and barley, this chromosome arm encodes ABA and GA opposite-regulated α-amylase genes [24], an ABA 8′-hydroxylase important for ABA catabolism and seed germination [25], and ABA responsive yet-uncharacterized gene(s) that are involved in dormancy [26] (Fig. 6).

### Application of ABA receptor antagonist, AS6, elicits few *Fg* DEGs but recapitulates the wheat DEGs induced by ABA and reduced by GA co-application

A synthetic ABA receptor antagonist would potentially serve as an excellent tool compound; however, AS6, an effective ABA receptor inhibitor in *Arabidopsis* [20], has not been established as a functional antagonist in wheat. *Fg* + AS6 enhanced the expression of 604 (8.8%) up-regulated and 904 (13%) down-regulated wheat DEGs elicited by *Fg* alone (Additional file 5: Figure S6A & D), while also suppressing 2685 (39%) up-regulated and 47 (0.66%) down-regulated wheat DEGs elicited by *Fg* alone (Additional file 5: Figures S6C & B). However, this treatment elicited few antagonistic DEGs with only 47 (1.5%) up-regulated and 22 (0.67%) down-regulated, when compared to those elicited by *Fg* + ABA (Additional file 5: Figure S6C and D; commonly elicited DEGs by these treatments may also be found in Additional file 5: Figure S6A and B and Figure S7). At this global transcriptomic level, evidence of AS6 antagonism on wheat ABA receptors was not clearly observed; therefore, comparisons of DEGs (Additional file 5: Figure S7) and DEFE patterns (Table 2) elicited by co-applied *Fg* + AS6 and *Fg* + ABA, compared to *Fg* alone, were conducted. Of the 316 DEGs up-regulated by *Fg* alone and further enhanced by *Fg* + ABA, 11 DEGs were suppressed by *Fg* + AS6 (highlighted in Additional file 5: Figure S6C). These included a γ-glutamyl phosphate reductase (TraesCS3B01G395900) and a eukaryotic peptide chain release factor subunit 1–1 (TraesCS1A01G235000; both genes adjusted *p* < 10^− 21^, Additional file 5: Figure S9) found in the DEFE patterns M111100 and M111101 (Additional file 5; Figure S8). Conversely, among the 279 DEGs down-regulated by *Fg* and further repressed by *Fg* + ABA, 29 DEGs were enhanced by *Fg* + AS6 (Additional file 5: Figure S6D). These included four DEGs that were repressed by ABA in the presence or absence of *Fg* challenge (*Fg* + ABA or ABA treatments) as evidenced by DEFE pattern M202202 and M222202 (Additional file 5: Figure S10). They include a BDX gene (TraesCS7B01G353600), transmembrane protein (TraesCS7A01G453100), leucine-rich repeat receptor-like protein kinase family protein (TraesCS3D01G040100), and nitrate transporter 1.1 (TraesCS1B01G225000; expression highly significantly different from *Fg* + ABA with adjusted *p* < 10^− 11^, Additional file 5: Figure S9). Furthermore, with respect to the *Fg* pathogen genes (Additional file 5: Figure S2A), *Fg* + AS6 repressed 26 DEGs including 11 putative oxidoreductases (*p* < 10^− 4^, Additional file 6 Tab ‘F1’, Additional file 7) and enhanced 17 DEGs including three putative O-glycosyl hydrolases (*p* < 4 × 10^− 3^, Additional file 6 Tab ‘F2’). This treatment also repressed 557 DEGs up-regulated by *Fg* + ABA and elicited no *Fg* + ABA- opposing effect (Table 1).

### Wheat host phytohormone biosynthesis and signaling gene expression is altered by *Fg* challenge and phytohormone application

Targeted analysis of transcriptomic changes to ‘Fielder’ phytohormone biosynthetic and signaling pathways elicited by *Fg* challenge and phytohormone application were performed based on local sequence alignment (BlastP) of known homologs against the wheat DEGs. Wheat challenged with *Fg* alone elicited DEGs in classic phytohormone defense responses including up-regulating SA (*PAL*), JA (*OPR3*) and ET (*ACS*) biosynthetic genes. In terms of signaling, SA (*NPR1*) and a repressor of JA (*JAZ*) were down-regulated, while JA (*OCRA3*) and ET (*ETR1*) signaling genes were up-regulated (Additional file 5: Figure S11, Additional file 1 Tabs ‘S2’ & ‘S3’). *Fg*-challenge also impacted wheat biosynthetic and signaling pathways of non-defense phytohormones (Fig. 6) with some mixed modulation of key contributors to the mevalonate and terpenoid biosynthesis pathways that feed into ABA, GA, CK and BR biosynthetic pathways. Finally, *Fg* challenge down-regulated ABA biosynthetic genes (*β -CRTZ*, *AOX*, and *A8H*), while up-regulating two GA biosynthetic genes (*GA20ox* and *GA2ox*) and down-regulating a GA signaling repressor (*DELLA*; Additional file 5: Figures S12 & S13).

**Fig. 7:**
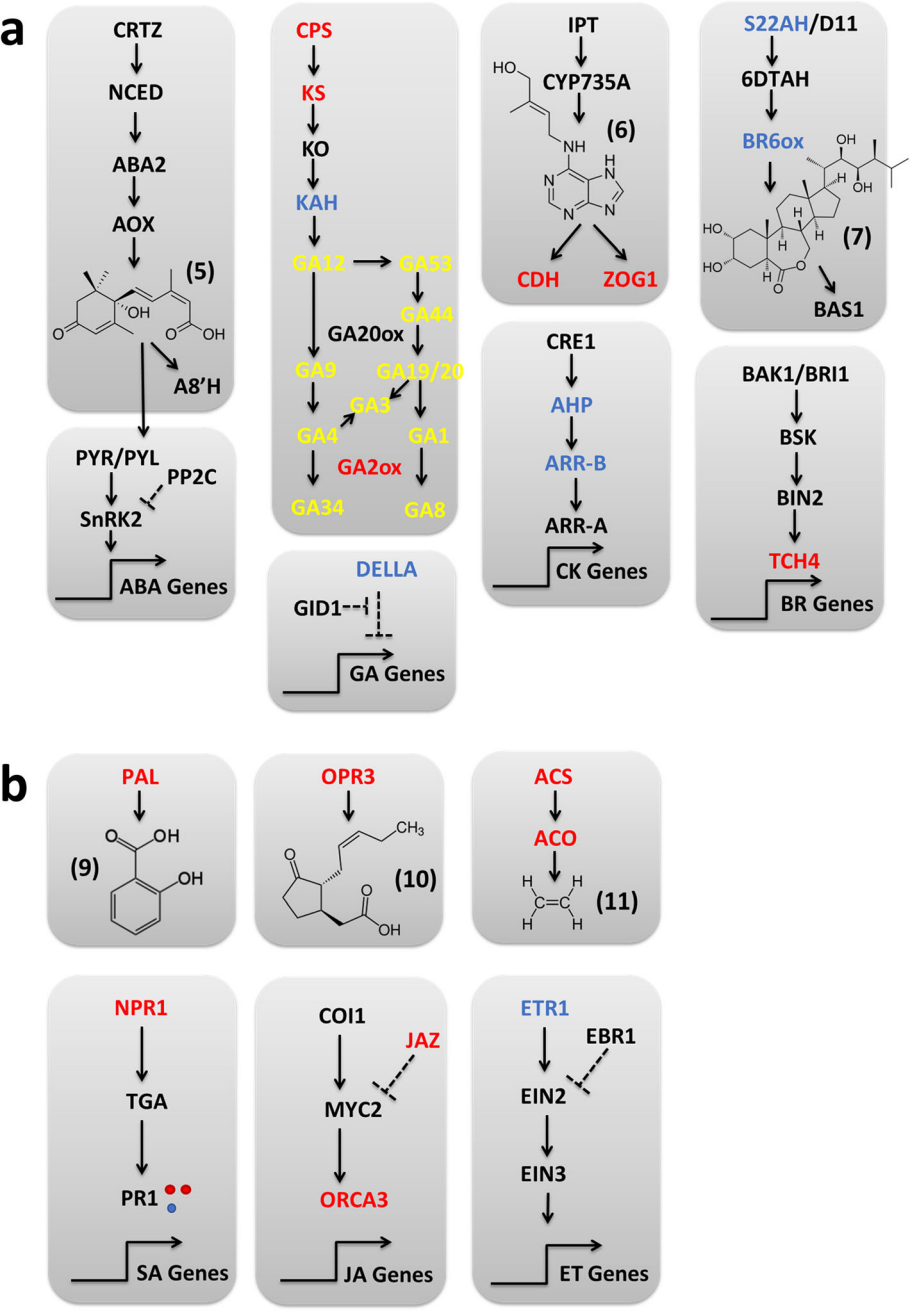
Phytohormone biosynthetic and signaling responses arising from co-application of AS6 at the time of *Fg* infection, with DEGs calculated compared to *Fg* alone. **a** ABA and other affected (non-classical defense) hormone pathways with chemical structures for (5) abscisic acid, (6) trans-zeatin and (7) brassinolide. GA metabolite acronyms are highlighted in yellow. **b** Affected classical defense pathways, with chemical structure for (9) salicylic acid (10) jasmonic acid and (11) ethylene. Legend details are otherwise the same as in Fig. 6. See Additional file 1, Tabs S2 and S3 for details

The co-applied condition of *Fg* + ABA elicited an up-regulation of wheat DEGs in the ABA (*HMGCR*, rate-limiting *NCED*, *A8H*), GA (*GA2ox* and *GA20ox*), ET (*ACO*) and JA (*OPR3*) biosynthetic pathways (Additional file 5: Figures S12 & S13). This induction of GA20ox and GA2ox wheat genes was consistent with the observed change in GA metabolites when comparing *Fg* challenged and *Fg* + ABA treated wheat cultivar ‘Fielder’ phytohormone profiles (Table 4). The *Fg* + ABA condition also yielded significant modulation of ABA signaling genes, including both suppression of multiple receptors and enhanced expression of multiple negative regulator PP2Cs, and up-regulation of SA (*PR1*), ET (*ETR1*), and BR (*TCH4*) signaling gene expression. These DEGs would be expected to reduce ABA signaling via two mechanisms and stimulate SA and ET signaling, with the latter two phytohormones both thought to have time-sensitive involvement in FHB defense [3, 6, 8, 9, 12]. Meanwhile, the co-applied *Fg* + GA suppressed the expression of GA biosynthetic genes (*KO* and *KAH*) and the negative regular *DELLA* involved in GA signaling. This condition also suppressed expression of ET (ETR1) and CK signaling genes (*A-ARR* and *AHP*) and BR biosynthetic genes (*S22AH* and *BR6ox*). The absence of an impact on wheat ABA signaling gene expression by the *Fg* + GA condition, and conversely GA signaling by the *Fg* + ABA condition, is generally consistent with the above noted suppression of *FUS3*, an important regulator at the intersection of ABA and GA crosstalk [27], under all conditions with *Fg* challenge (Additional file 5: Figure S1). Finally, the co-applied condition of *Fg* + AS6 up-regulated wheat DEGs in SA (*PAL*), JA (*OPR3*), ET (*ACS* and *ACO*), GA (*CPS*, *KS*, and *GA2ox*), and CK (*CDH* and *ZOG1*) biosynthesis while repressing genes in BR biosynthesis (*S22AH* and *BR6ox*). This condition also impacted wheat signaling gene expression with induction of SA (*NPR1*), JA (repressive *JAZ* and *ORCA3*), and BR (*TCH4*) while repressing ET (*ETR1*), GA (the repressive *DELLA*), and CK (*CDH* and *ZOG1*) genes alone (Fig. 7). Interestingly, *Fg* + AS6 did not elicit changes to ABA metabolic or signaling pathways nor antagonistic changes to phytohormone pathways when compared to *Fg* + ABA (except for DEGs corresponding to ACS in the ET pathway).

**Table 4:** ABA and GA phytohormones and their metabolites detected in ‘Fielder’ spikes upon *Fg* challenge. Phytohormones and their associated metabolite levels were detected in ‘Fielder’ spikes (normalized to dry weight (DW)) inoculated with *Fg* in the absence (*Fg*) or presence of either ABA (*Fg* + ABA) or GA (*Fg* + GA). The ABA metabolites phaseic acid and dihydrophaseic acid are abbreviated PA and DPA, respectively, while undetected metabolites are denoted ‘ND’. Phytohormone changes were evaluated with one-way ANOVA with Dunnett post-hoc comparisons (* *p* ≤ 0.05, ** *p* ≤ 0.01, *** *p* ≤ 0.001, **** *p* ≤ 0.0001)

Metabolite (ng/g DW)	Treatment		
	***Fg***	***Fg + ABA***	***Fg + GA***
ABA	143 ± 37.4	32,800 ± 5500 ****	75.1 ± 21.6
DPA	205 ± 274	49,400 ± 10,600 ***	58.4 ± 39.8
PA	91.0 ± 88.8	55,500 ± 18,100 **	34.6 ± 17.9
7′-OH ABA	155 ± 51.1	7900 ± 2070 ***	77.6 ± 21.9
*neo-PA*	23.0 ± 8.4	975 ± 216 ***	4.9 ± 1.2
*trans-ABA*	42.0 ± 34.4	1460 ± 769 *	7.6 ± 2.4
GA1	ND	6.7 ± 1.5	249 ± 22.3
GA3	37.0 ± 16.7	24.8 ± 13.2	343 ± 13.4 **
GA8	15.0 ± 5.7	11.2 ± 2.3	48.4 ± 9.8 *
GA19	19.0 ± 7.2	19.0 ± 2.5	25.2 ± 6.0
GA20	ND	ND	6.3 ± 0.7
GA44	ND	10.0 ± 0.6	5.1 ± 0.5

### Generation of a consensus model of wheat transcriptomic responses to *Fg* challenge

In this work, the comparative transcriptomic responses of ‘Fielder’ are characterized; however, it remains unclear what proportion of, or trends in, this high-quality ‘Fielder’ transcriptome data have the greatest biological implications, especially considering disagreements between previous reports [6]. The results identified herein were compared with the four other FHB susceptible genotypes ‘Roblin’ [10, 11, 28], ‘Shaw’ [8], ‘Stettler’ and ‘Muchmore’ [9] to identify the strongest, consistent responses that may contribute to a consensus model of FHB infection. Findings are discussed if three or more genotypes reported significant expression changes in spikelet tissue. First, of the approximately 2600 ‘Fielder’ key hub genes identified upon *Fg* challenge here (Additional file 2 Tab ‘WheatDEGs’@Col-P), many were highly regulated by all genotypes. These changes include down-regulation of fatty acid hydroxylase superfamily (13 DEGs with mixed regulation of 5 additional DEGs), photosystem II 22 kDa (3), PISTILLATA-like MADS-box transcription factor (2) and D-glycerate dehydrogenase/ hydroxypyruvate reductase and up-regulation of C2 domain-containing protein-like (5 DEGS with mixed regulation of 4 additional DEGs) and yellow stripe-like transporter 12 (4) (Additional file 8 Tab ‘Hub-Genes’). Secondly, among the genes involved in the phytohormone biosynthetic pathways (Additional file 1, Tab S2), *Fg* challenge up-regulated ET (three ACS synthase genes), JA (OPR3), and SA (PAL) biosynthetic DEGs while also up-regulating JA (ORCA3) and auxin (CH3) signaling DEGs (Additional file 1, Tab S3). Pathogen treatment consistently repressed auxin biosynthesis (IPMO and ADH). There is also moderate agreement within the other comparative reports describing the up-regulation of jasmonate ZIM-domain (7) and down-regulation of pectinesterase (4) (Additional file 8, Tab Hub-genes). Third, an overlapping set of DEGs involved in aromatic and nitrogen metabolism are down-regulated and include a CTP synthase, DNA-directed RNA polymerase, and phenylalanine ammonium lyase along with mixed changes among several other DEGs (Additional file 8 Tab ‘aromatic’ and ‘Cell N’). Thirteen additional up-regulated phenylalanine ammonium-lyase DEGs involved in cinnamic acid biosynthesis demonstrate substantial agreement between genotypes (Additional file 8 Tab ‘cinnamic’). Fourth, a consensus pattern emerged herein with GA treatment down-regulating and *Fg* up-regulating sets of DEGs. These DEGs were involved in heme (hemoglobin and cytochrome P450), oxidoreductase (cytochrome P450, polyphenyl oxidase, and aldehyde oxidase), and hydrolase (α/β hydrolase superfamily) activities (Additional file 8 Tabs ‘Heme’, ‘oxidoreductase’, and ‘hydrolase’). Finally, there is moderate agreement in the other four comparative genotypes reporting up-regulation of hydrolase (β-amalase, β-fructofuranosidase, β-glucosidase, pectin acetylesterase, pectinesterase, and ribonuclease) and oxidoreductase (protochlorophyllide reductase, laccase, and respiratory burst oxidase) related DEGs (Additional file 8 Tabs ‘hydrolase’ and ‘oxidoreductase’).

## Discussion

Comparative transcriptomic studies continue to be insightful in understanding general mechanisms modulating host resistance and susceptibility when challenged with *Fg* (reviewed in [6]). An encouraging flurry of recent publications emphasize roles for non-classical defense phytohormones in both resistance and susceptibility [7–11], suggesting new focused alternative mechanisms underlying the host-pathogen interaction. To date, these studies have highlighted variable responses between examined cultivars, an aspect which is further complicated by recent evidence suggesting that non-classical defense phytohormones are also biosynthesized by *Fg* itself [14, 16, 18, 19]. In this light, the expression of a putative fungal ABA biosynthetic cytochrome P450 homolog under all conditions containing *Fg* examined herein is noted (Additional file 5: Figure S2B), lending further support to the role for fungal-derived ABA as an effector of infection, as has been proposed previously [14, 29].

### Assimilating ‘Fielder’ transcriptomic changes by comparing patterns of explicit features

The wheat whole-transcriptome analyses reported here were aimed at characterizing treatment-elicited changes in ‘Fielder’ and *Fg* gene expression by distilling the tens of thousands of observations into distinct strings of DEG permutations using DEFE. These combinations of explicit characteristics may then be used to understand the relationship between gene regulation and phenotype, especially when considering multiple treatment types [22]. DEFE descriptors quantifying the veritable impact of *Fg* challenge on wheat gene expression, highlighted abundant ‘Fielder’ expression changes elicited by ABA or GA application alone, and described the unexpected DEG overlaps of *Fg* + AS6 with *Fg* + GA (86%) and *Fg* + ABA (23%). Furthermore, by comparing individual DEFE descriptors from single phytohormone treatments with the intersection of several treatments (e.g., ABA∩*Fg*∩[*Fg* + ABA]), DEGs elicited by ABA or GA alone were shown to be common in the co-application samples in 91 and 83% of DEGs, respectively. By combining DEFE with topology overlap and gene association network analyses, more than 9500 ‘Fielder’ DEGs were characterized as *Fg*-related, where the most highly enriched genes are putatively involved in stress, cell structural integrity, membrane/lipid homeostasis, or molecular transport. As expected in ‘Fielder’ spikelets, many of these enriched genes are putatively involved in flowering [30–32]. Finally, *FUS3* was identified as one notable gene in the network, having greater than 2000 connections. The *FUS3* ortholog in *Arabidopsis thaliana* (AT3G26790) is known to be involved in positive regulation of ABA biosynthesis and negative regulation of GA biosynthesis [27]. As *FUS3* gene expression was strongly suppressed across all *Fg-*challenged conditions, the presence of the pathogen likely dysregulates ABA and GA crosstalk and may decouple their often-oppositional biological phenotypes. In additional to biosynthesis, the disruption of both ABA and GA signaling has been observed in the presence of *Fusarium* virulence factors [33], suggesting there may be multiple mechanisms used by the pathogen to dysregulate ABA and GA related plant responses.

### Wheat transcriptomic responses to Fg challenge include ubiquitous traditional defense responses and more nuanced changes in non-classical defense phytohormones

*Fg*-challenge of wheat varieties elicits an incontestable shift of host gene expression with noted variations in the expression of phytohormone biosynthetic and signaling pathway genes [8–11, 34]. When investigating ‘Fielder’ in this work, *Fg* challenge up-regulated both SA and JA biosynthetic pathways, notably with markers of late SA signaling highlighted, consistent with previous reports [8, 34]; while mixed directions in differential gene expression of ET biosynthesis are juxtaposed to induction of one ET receptor gene, together suggesting ET pathways are being ‘primed’ for response. Beyond these classical defenses, *Fg*-challenge also elicited mixed expression changes of biosynthetic and signaling pathways of ABA, GA, IAA, BR, and CK where GA and IAA biosynthetic genes were up-regulated, GA and BR signaling genes were down-regulated, and ABA signaling genes were up-regulated.

When investigating cereal-*Fusarium* interactions using transcriptomic approaches, a variety of phytohormone responses have been observed which often do not agree between reports [6]. Wang et al. [9] may be most comparable to this work; therefore, a direct comparison may be more appropriate than a field-wide discussion in which little consensus is noted. Both works characterize up-regulation of genes involved in SA biosynthesis and SA, JA, ET, and ABA signaling. However, here we report increased JA and mixed ET biosynthetic gene expression changes, while Wang et al. characterize susceptible lines down-regulating JA and up-regulating ET biosynthetic genes with a contrasting increase in JA phytohormone concentration. Based on the consensus model of early biotrophic SA followed by later stage necrotrophic JA/ET responses [6, 8, 9], these differences may suggest that ‘Fielder’ tissues were analyzed while still transitioning to the later JA (increased biosynthesis) / ET (‘primed’ biosynthesis) response, whereas Wang et al.’s ‘Settler’ and ‘Muchmore’ varieties were characterized at a later infection state. This hypothesis is consistent with the ‘Fielder’ transcriptome being derived from early-stage at 24 h post inoculation (hpi), challenged spikelets while the ‘Settler’ and ‘Muchmore’ transcriptomes were derived from three pooled whole spikes with singly challenged spikelets (4 days post inoculation).

### ABA induces wheat DEGs observed in Fg infected tissues and may promote disease severity by misregulating phytohormone defense response and cell wall fortification mechanisms

Unlike the model of early SA and later JA/ET responses triggered in wheat challenged with *Fg* [6, 8, 9], the gene expression changes upon application of individual phytohormones to wheat has not been coalesced into a single model. Perhaps the most comprehensive study of the differential gene expression has been the work of Qi et al. [11] where wheat spikelets were treated with IAA, GA, ABA, ET, CK, SA or JA (methyl jasmonate) and resultant DEGs characterized by microarray. Surprisingly, there were many contradictory findings when comparing the findings of Qi et al. and the transcriptomic changes presented here, namely in the number of DEGs identified per treatment and the relative proportionality between up- and down-regulated DEGs within a given treatment. These differences may be in part due to comparison between pooled wheat spikes vs treated spikelets or the comparison between two different genotypes, ‘Roblin’ vs ‘Fielder’. It may also be method-dependent where wheat microarray analysis is limited to 50,000–60,000 genes [11, 34]; and additional stringency criteria must be applied to properly control for complications such as cross-hybridization and limited dynamic assay range (as reviewed in [35]). Despite these differences, both studies report a significant overlap between DEGs elicited by *Fg* challenge and ABA application as well as the ABA-dependent regulation of polyphenolic compound-related gene expression. Herein, exogenously applied ABA alone yielded a general repression in ‘Fielder’ where 57% of up-regulated and 75% of down-regulated genes were also elicited by *Fg* challenge. Most of the genes (> 90%) significantly modified by ABA alone and *Fg* alone were also significantly modified in the co-applied *Fg* + ABA condition, highlighting the relevance of findings from studies of ABA-alone with respect to understanding the interaction of ABA with *Fg* infection. However, ABA treatment outcomes are not entirely overlapping with those elicited by either *Fg* or *Fg* + ABA. One notable case is the ABA down-regulated but *Fg* up-regulated polyphenolic compound-related genes. Phenylpropanoid metabolism is involved in the biosynthesis of lignins, cinnamic acids, and flavonoids that are in turn used for cell wall fortification and *Fg* defense [36–40]. FHB resistant wheat and durum varieties have been shown to have key anatomical differences, improved cell wall structure including the additional lignin deposition, and greater accumulation of the cinnamic acid metabolite *p*-coumaric acid in infected tissues as compared to susceptible varieties [37, 41–43]. Furthermore, some polyphenolic compounds have documented anti-fungal activities [44, 45]. In addition to the ABA alone treatment, the co-applied *Fg* + ABA treatment also highlighted altered physical defense responses and non-classical defense phytohormone signaling, specifically suppressing IAA and enhancing BR signaling. IAA signaling suppression is consistent with heightened classical defense responses and has been detected in susceptible near isogenic lines of the 2DL QTL, compared to more resistant lines [46, 47]. Enhanced BR signaling included the expression of a cell wall re-modelling xyloglucan endotransferase gene (*TCH4*) that may contribute to FHB susceptibility through inappropriately regulated cell wall modification. Together, wheat transcriptomic changes elicited within ABA treatment groups suggests that this phytohormone promotes FHB disease severity (this work, [14, 15]) by inducing wheat gene expression changes necessary for *Fg* infection including misregulating classical defense phytohormone signaling and physical defense mechanisms, such as plant cell wall fortification and polyphenolic metabolism.

### GA induces global metabolic shifts in both the wheat host and Fg pathogen

Historically, both genetic and chemical approaches have been applied to modulate GA metabolism for the improvement of agronomic wheat characteristics [48, 49]. It remains to be determined whether targeting GA metabolism could be a reasonable strategy for controlling FHB, based on the observation that GA-regulated wheat anthesis and plant height are strongly correlative with infection and disease severity [1, 50–52]. Based on the transcriptomic profiles of wheat treated with diverse phytohormones, it was determined that ABA and GA elicit the strongest transcriptional antagonism of any phytohormone pair, with approximately 40% of their DEG being antagonistic [11]. These findings are also in agreement with previous reports of ABA promoting and GA suppressing FHB disease severity [14, 15]. Here, exogenously applied GA alone resulted in a relatively even mix of up-regulated and down-regulated ‘Fielder’ genes including nearly 500 DEGs that directly opposed those elicited by *Fg* alone. GO enrichment suggest that these oppositional effects included down-regulated secondary metabolism and defense response genes, coincident with up-regulated primary metabolic processes. These GA derived DEGs describe a broader metabolic switch with moderate overlap compared to the primary carbohydrate, TCA, and nitrogen metabolic changes elicited by DON treatment [53]. It is also interesting to note that *Fg* transcriptionally regulates its own metabolism when transitioning from a biotrophic to necrotrophic state during the first 48 h post infection [54]. In Buhrow et al., [15], the effect of *Fg* + GA co-application on *Fusarium* gene expression was characterized with this phytohormone eliciting DEGs broadly within amino acid, carbohydrate, and lipid metabolism and specifically regulating five genes in inorganic nitrogen or amino acid nitrogen metabolism. This coupling of GA and nitrogen metabolism has been previously described in the related *Fusarium moniliforme* [55]. Therefore, these findings suggest that GA may limit FHB severity through global metabolic changes on both the wheat host and *Fg* pathogen rather than through a select few defense pathways.

### The Fg + AS6 treatment functions independently of ABA-signaling to antagonize ‘Fielder’ gene expression elicited by ABA∩Fg∩[Fg + ABA] treatments

Co-application of AS6, a rationally designed ABA analog with a well-characterized ability to disrupt ABA receptor interactions in dicots [20], was investigated for its ability to elicit antagonistic transcriptomic responses compared to ABA. Notably ABA receptor functionalities in the wheat variety ‘Thatcher’ have been characterized previously [56]. In Fielder, *Fg* + AS6 elicited robust antagonist responses compared to DEGs elicited by ABA∩*Fg*∩[*Fg* + ABA]. Specifically, this treatment mitigated the up-regulation of γ-glutamyl phosphate reductase and eukaryotic peptide chain release factor subunit 1–1 genes elicited by ABA∩*Fg*∩[*Fg* + ABA]. The expression of γ-glutamyl phosphate reductase has been tied to abiotic stress response [57]; while eukaryotic peptide chain release factor subunit 1–1 has been tied to plant growth and development including appropriate cell-wall lignification [58]. *Fg* + AS6 also robustly up-regulated expression of a leucine-rich repeat receptor-like protein kinase (LRRKs) and nitrate transporter 1.1 that were down-regulated by ABA∩*Fg*∩[*Fg* + ABA] treatments. LRRKs have been linked to FHB resistance in durum [50]; while nitrate transporter 1.1 has been suggested to function as a bridge between phytohormone responses during pathogen stress (reviewed in [59, 60]). As all the robust *Fg* + AS6 responses can be tied to plant stress responses, and in some cases even FHB and phytohormone pathways, AS6 may in fact promote FHB resistance when applied in an appropriate developmental and sustained manner.

### Agreement between transcriptomic responses derived from several genotypes support a consensus model of the wheat-Fg interaction

Previous transcriptomics reports of *Fg*-challenged wheat highlighted up-regulated basal defenses, antagonism of pathogen-mediated modulation of phytohormone pathways, and classical phytohormone defense signaling [61–65] with an early biotrophic SA followed by later stage necrotrophic JA/ET responses [6, 8, 9]. To additively contribute to this consensus model, differential gene expression were compared among five FHB susceptible wheat genotypes. Generally ubiquitous up-regulation of C2 domain-containing protein-like, jasmonate ZIM-domain, yellow stripe-like transporter 12, phenylalanine ammonium-lyases (cinnamic-acid metabolism) and down-regulation of hydrolases that putatively cleave fatty acids, pectins, ribose, and sugar moiety substrates were observed. Interestingly, ABA treatment also regulates DEGs involved in cell wall-modifying polyphenolic and cinnamic acid metabolism and a saccharide hydrolase/ transferase, xyloglucan endotransferase. Meanwhile, GA treatment responses are targeted to metabolic processes like those elicited by DON treatment (discussed previously).

## Conclusions

A comparative transcriptomic approach was applied in this work to elucidate the mechanisms by which ABA and GA affect wheat ‘Fielder’ FHB severity. Gene expression differences reported herein emphasize the vast impact of pathogen challenge and specifically highlight genes involved in defense response, cell structural integrity, molecular transport, and membrane/lipid metabolism as *Fg-*responsive. *Fg* challenge notably down-regulated expression of a key regulator of ABA and GA crosstalk, *FUS3*, supporting independent mechanisms by which ABA promotes and GA reduces FHB disease severity. Transcriptomic profiles presented herein emphasized that ABA co-application promotes disease by eliciting responses common to those elicited by the pathogen, misregulating defense responses by further exacerbating gene expression, and altering expression of cell wall fortification mechanisms. Unexpectedly, AS6 did not transcriptionally antagonize ABA signaling or biosynthesis in ‘Fielder’ but opposed other ABA- and *Fg-*elicited responses, highlighting potential applications of ABA antagonists in future FHB research and disease mitigation efforts. In contrast, GA co-application elicited transcriptomic responses with diverse metabolic implications for both the ‘Fielder’ host and *Fg* pathogen. Such metabolic reprogramming may result in a less susceptible host and/or less virulent pathogen, especially at a time co-incident with *Fg*’s biotrophic to necrotrophic lifestyle transition, ultimately reducing disease severity. Finally, the biological implications of these transcriptome responses on the wheat-*Fg* interaction were evaluated by assessing common findings across five wheat genotypes, additively contributing to a limited consensus model of disease response and severity.

## Methods

### Chemicals, phytohormones and *Fg* inoculum preparation

Gibberellin A3 was purchased from Sigma-Aldrich (St. Louis, MO). The National Research Council Hormone Profiling Facility provided (+)-ABA, while 3′-hexasulfanyl-(+)-ABA was synthesized as described in Takeuchi et al., [20] and provided by Kenneth Nelson and Suzanne Abrams at the University of Saskatchewan. Phytohormone stocks were solubilized in deionized water as sodium salts by 1.0 N NaOH titration and stored at − 20 °C in amber vials. Working solutions were made in deionized water and pH was adjusted to 7.0 ± 0.05. *Fg* GZ3639 [66] was propagated on potato dextrose agar (PDA; Sigma-Aldrich) at 25 °C for 5 days. To obtain spores, carboxymethylcellulose (CMC) liquid media (1.5% CMC (Sigma), 0.1% NH_4_NO_3_, 0.1% KH_2_PO_4_, 0.05% MgSO_4_·7H_2_O, and 0.1% yeast extract) was inoculated with a marginal 5 mm square PDA plug and grown for 5 days at 27 °C, shaking at 170 rpm. Macroconidia were isolated by filtering through one layer of cheese cloth and 25 μm Miracloth filter (EMD Millipore; Billerica, MA), washed three times with sterile water, and quantified using a haemocytometer and light microscopy.

### Propagation of plants, pathogen-challenge +/− co-applied phytohormones, and phytohormone profiling

*T. aestivum* variety ‘Fielder’ [67], obtained from Dr. Mark Jordan (Agriculture and Agri-Food Canada, Morden, MB, Canada), was grown in Sunshine^R^ Mix 8 (Sungrow Horticulture, Agawam, MA) and maintained in climate-controlled chambers with a 16 h photoperiod, at 25 °C followed by 8 h of dark at 16 °C every day. Plants were watered as needed, fertilized biweekly with 20–20-20 (N-P-K) and trimmed regularly prior to infection with *Fg* to remove yellowing material or material with any signs of disease. At the two-leaf stage plants were treated with Intercept™ (Bayer Crop Science, Calgary, AB) as an aphid preventative, as previously described [15, 34]. During anthesis, florets from a central spikelet (destined for RNA sequencing) or entire spikes (destined for phytophormone profiling) were point inoculated with 10 μL of 5.0 × 10^4^ *Fg* GZ3639 macroconidial suspension or deionized water (mock). For phytohormone application or co-application, this inoculum contained 1.0 mM ABA, GA3, or AS6. To promote infection, wheat plants were transferred to climate-controlled chambers with misting to achieve 90% humidity for 24 h.

Phytohormone content in individual ‘Fielder’ spikes was determined at 24 hpi. For each condition (*Fg*, *Fg* + ABA, *Fg* + GA), one spike on five different plants was treated. Individual spikes served as a single tissue sample (5 spikes = 5 biological replicates with no tissue pooling). These individual spikes were flash frozen and ground in liquid nitrogen. Phytohormones were extracted and quantified by UPLC/ESI-MS/MS at the National Research Council of Canada in Saskatoon, Canada, as described in [68–72]. Phytohormone content differences were analyzed with one-way ANOVA with Dunnett post-hoc comparisons using GraphPad Prism 6 (GraphPad Software, Inc. La Jolla, CA).

### RNA sequencing, data processing, and differential expression analyses

A total of seven ‘Fielder’ spikelet treatments (*Fg*, MT, ABA, GA, *Fg* + ABA, *Fg* + GA. *Fg* + AS6) were analyzed at 24 hpi, each consisting of five biological replicate central spikelet tissues. Total RNA was extracted from these central spikelets (35 samples in total), purified, quantified, and sequenced as described previously [15]. In short, RNA was purified using the RNeasy Plant Mini Kit (Qiagen, Mississauga, ON) using an on-column DNaseI treatment (Qiagen). RNA quality was evaluated using NanoDrop ND-8000 (NanoDrop, Wilmington, DE) and agarose gel electrophoresis. RNA library construction was completed using 1.0 μg total RNA and the TruSeq RNA Sample Prep Kit v2 (Illumina, SanDiego, CA). Library quality (RNA integrity number 8.9 ± 0.38, Supplemental S1) was confirmed using the 2100 Bioanalyzer (Agilent Technologies Inc. Santa Clara, CA) equipped with a DNA 1000 chip. Library concentrations were determined by quantitative polymerase chain reaction (qPCR) using the KAPA SYBR FAST ABI Prism qPCR Kit (Kapa Biosystems, Wilmington, MA) and the StepOnePlus Real-Time PCR System (Applied Biosystems, Foster City, CA). RNA samples were multiplexed at a sequencing depth of five libraries per lane. Equimolar concentrations of the libraries were pooled and a final concentration of 12 pM was used for clustering in cBOT (Illumina) flowcell lanes. The samples were then sequenced (2 × 101 cycles, paired end reads) on the HiSeq2500 (Illumina) using the TruSeq SBS Kit v3-HS 200 cycles Kit (Illumina). The raw RNA-seq reads (available at GEO GSE137895) were preprocessed by trimming the adaptor sequences, filtering low-quality reads (Phred Score ≤ 20 [73]), and eliminating short reads (length ≤ 20 bps) using software package FASTX-toolkit (default settings) [74]. After filtering, barcode and adaptor removal, an average of 27 million paired reads per sample was retained for subsequent read mapping through the RNA-seq data processing procedures. The IWGSC RefSeq v1.0 complete reference genome and corresponding annotation v1.0 were used as reference for the analysis of wheat RNA-seq data [21]. Following the recommendation of IWGSC, the chromosome partitioned version (161010_Chinese_Spring_v1.0_pseudomolecules_parts.fasta) was used and the gff3 file was reformatted accordingly. Only the high confidence gene models were considered in the mapping process. The *Fg* reference genome (*Fusarium graminearum* str. PH-1) was obtained from EnsemblFungi (http://fungi.ensembl.org/ Release 35). Wheat and *Fg* genomes and annotation data from both species were combined into a single reference [75]. This combined reference genome contains 124,935 gene models, 14,145 from *Fg* and 110,790 from wheat. The cleaned RNA-seq reads in each sample were mapped using STAR v2.5.3a (default settings) [76] to generate gene-level read counts. DESeq2 [77] was used for data normalization and subsequent differentially expressed gene (DEG) analysis for each pairwise comparison. Normalized read counts along with log_2_ fold change and *p*-value, and adjusted *p*-values based on Benjamini and Hochberg procedure [78], were provided for downstream data analysis. The bioinformatics R script is available at the Github repository (https://github.com/DT-NRC/RNA-Seq-DataProcessingProcedure).

### Data reduction and partitioning

The output data from DESeq2 [77] was reduced in size to a set of differentially expressed genes. We applied the criteria of absolute log_2_FC ≥ 1, adjusted *p* ≤ 0.01, and one of the pair of compared samples must be significantly expressed (i.e ≥ 10 reads). Six informative pairwise comparisons between each treatment and MT and three pairs between co-application of *Fg* with a phytohormone and *Fg* alone and a comparison between co-applications of *Fg* with ABA and with AS6 (Fig. 1c, Table 1) were performed. The differential expression feature extraction method (DEFE [22];) was applied to partition the DEGs into various feature patterns. We compiled the six treatments compared to MT as one series of “M” patterns: M($\frac{ABA}{MT}$, $\frac{GA}{MT}$, $\frac{Fg}{MT}$, $\frac{Fg+ ABA}{MT}$, $\frac{Fg+ AS6}{MT},$ $\frac{Fg+ GA}{MT}$) (Additional files 2 & 3 Tab “DEFE stats”). For example, in the pattern M201110, at each comparison, “0” denotes no change, “1” for up- and “2” for down-regulation; this example pattern means a gene is down-regulated by ABA alone, has no significant change with respect to GA, whether applied with or without *Fg*, but is up-regulated by *Fg* alone, *Fg* + ABA and *Fg* + AS6. Similarly, we compiled the three combinations of respective phytohormones with *Fg* infection as compared with *Fg* infection alone in a series “F” patterns: F($\frac{Fg+ ABA}{Fg},$ $\frac{Fg+ AS6}{Fg},$ $\frac{Fg+ GA}{Fg}$) (Additional file 2 Tab “DEFE stats”).

### Clustering, correlation, network, gene ontology enrichment, and orthology analyses

These analyses were done following the same methods described in [8]. Briefly, detailed methods for clustering, correlation and network analyses were provided in WGCNA R package [23] with the same modification as described in [8]; gene ontology enrichment analysis was performed using GOAL [79]; and reciprocal best hit BlastP was used for orthology determination.

### Phytohormone pathway gene identification and analyses

BlastP analyses of representative biosynthetic and signaling query sequences were performed against the DEGs derived in this study based on the Kyoto Encyclopedia of Genes and Genomes (KEGG) Database (cut off > 50% amino acid sequence identity). This yielded significant DEGs (log_2_FC > 1.5 or < − 1.5; adj *p* < 0.01; to highlight the more highly modified) covering the spectrum of plant phytohormone biosynthesis and signaling pathways (Additional file 1 Tabs ‘S2’ & ‘S3’ respectively).

## Supplementary Information

**Additional file 1: supplementary tables.** This file contains four tables: (S1) RNA-seq read mapping statistics, (S2) ‘Fielder’ phytohormone biosynthetic pathways are affected by co-application of hormones with *Fg* challenge, compared to hormone- or *Fg*- challenge alone, (S3) ‘Fielder’ phytohormone signalling pathways are affected by co-application of hormones with *Fg* challenge, compared to hormone- or *Fg*-challenge alone, and (S4) distribution of phytohormone pathway genes over the chromosome arms.

**Additional file 2. **Differentially Expressed Wheat genes. This file contains three worksheets: (1) a list of 26,001 wheat DEGs, their expression values, cluster membership, gene network property, differential expression (log_2_FC and adj *p* values), DEFE pattern, and correlation analysis, etc.; (2) DEFE statistics, number of genes belong to each DEFE pattern, full lists of Tables 2 and 3; (3) correlation analysis of the 58 clusters.

**Additional file 3. **Differentially Expressed Genes of *Fusarium graminearum.* This file contains two worksheets: (1) a list of 4872 *Fg* DEGs, including their expression values, differential expressions (log_2_FC and adj *p* values), and DEFE pattern.

**Additional file 4.** Enrichment index of various genes with their membership in various groups including DEGs, FHB-related genes, top 1% gene Network, and Key hub genes.

**Additional file 5.** 13 Supplementary Figures.

**Additional file 6.** Three series of Gene Ontology analyses in 32 worksheets plus a summary note worksheet: (1) FD series: ten lists of wheat genes down-regulated by *Fg*; (2) FU series: 19 lists of wheat genes up-regulated by *Fg*; (3) F series: three groups of *Fg* genes differentially expressed as affected by AS6 and others.

**Additional file 7.** Twenty-six up-regulated genes by *Fg* alone were suppressed by *Fg* + AS6.

**Additional file 8.** Comparisons across five wheat genotypes susceptible to FHB. This file contains seven worksheets: (1) network hub genes; (2) genes involved in the aromatic metabolism; (3) genes involved in the cellular nitrogen metabolism; (4) genes involved in the cinnamic acid biosynthesis; (5) genes involved in the heme activity; (6) genes involved in the oxidoreductase activity; and (7) genes involved in the hydrolase activity.

## Data Availability

The datasets generated and/or analyzed during the current study are available in the GEO repository, GSE137895.

## Abbreviations

ABA: Abscisic acid
BR: Brassinosteroid
CK: Cytokinin
DEFE: Differential expression feature extraction
DEG: Differential gene expression
*Fg*: *Fusarium graminearum*
FHB: Fusarium head blight
ET: Ethylene
GA: Gibberellic acid
IAA: Indole acetic acid (auxin)
JA: Jasmonic acid
SA: Salicylic acid
